# Strengthening health systems in low-income countries by enhancing organizational capacities and improving institutions

**DOI:** 10.1186/s12992-015-0090-3

**Published:** 2015-02-12

**Authors:** Robert Chad Swanson, Rifat Atun, Allan Best, Arvind Betigeri, Francisco de Campos, Somsak Chunharas, Tea Collins, Graeme Currie, Stephen Jan, David McCoy, Francis Omaswa, David Sanders, Thiagarajan Sundararaman, Wim Van Damme

**Affiliations:** Department of Health Sciences, Brigham Young University, Provo, UT 84602 USA; Department of Global Health and Population, Harvard School of Public Health, Harvard University, 665 Huntington Avenue, Boston, MA 02115 USA; Vancouver Coastal Health Research Institute, 6975 Marine Drive West, Vancouver, BC V7W 2 T4 Canada; Health Systems Action Network, K-57 A, Sheikh Sarai Phase -II, New Delhi, 110017 India; Nucleus of Research in Public Health, Faculdade de Medicina da UFMG, Avenida Alfredo Balena, 190, Belo Horizonte, MG Brasil; National Health Foundation, Building 1, 1st Floor, Office of the Permanent Secretary, Ministry of Public Health, Tivanond Road, Nonthaburi, 11000 Thailand; World Medical Association, 13 Chemin du Levant, Bâtiment A, le John Keynes, 01210 Ferney-Voltaire, France; Warwick Business School, The University of Warwick, Coventry, CV4 7AL UK; George Institute for Global Health, Level 10, King George V Building, 83-117 Missenden Rd, PO Box M201, Camperdown, NSW 2050 Australia; Queen Mary University, London, Blizard Institute, Barts and The London School of Medicine and Dentistry, 4 Newark Street, London, E1 2AT England; African Centre for Global Health and Social Transformation, Plot 13 B Acacia Avenue, P.O.Box 9974, Kololo, Kampala Uganda; School of Public Health, University of Western Cape, South Africa, Private Bag X17, Bellville, 7535 Republic of South Africa; National Health Systems Resouce Centre, NIHFW Campus, Baba Gangnath Marg, Munirka, New Delhi, 110067 India; Institute of Tropical Medicine, Antwerp, Belgium and University of Western Cape, Sint-Rochusstraat 2, 2000 Antwerpen, Belgium, South Africa

**Keywords:** Complex adaptive systems, Health systems, Complexity, Capacity, Systems thinking, Low-income countries, Institutions, Organizational studies

## Abstract

**Background:**

This paper argues that the global health agenda tends to privilege short-term global interests at the expense of long-term capacity building within national and community health systems. The Health Systems Strengthening (HSS) movement needs to focus on developing the capacity of local organizations and the institutions that influence how such organizations interact with local and international stakeholders.

**Discussion:**

While institutions can enable organizations, they too often apply requirements to follow paths that can stifle learning and development. Global health actors have recognized the importance of supporting local organizations in HSS activities. However, this recognition has yet to translate adequately into actual policies to influence funding and practice. While there is not a single approach to HSS that can be uniformly applied to all contexts, several messages emerge from the experience of successful health systems presented in this paper using case studies through a complex adaptive systems lens. Two key messages deserve special attention: the need for donors and recipient organizations to work as equal partners, and the need for strong and diffuse leadership in low-income countries.

**Summary:**

An increasingly dynamic and interdependent post-Millennium Development Goals (post-MDG) world requires new ways of working to improve global health, underpinned by a complex adaptive systems lens and approaches that build local organizational capacity.

## Background

The current interest in health systems strengthening (HSS) provides an opportunity for the global health community to focus on building organizational capacity by improving institutions in low-income countries [1]. Reductionist approaches to improving health that emphasize short-term goals have too often undermined local health systems by neglecting to strengthen local organizations. For example, routing funds from global health initiatives through external organizations at the expense of local ones can lead to fragmented delivery systems, duplication of services, high transaction costs, inefficiencies, and missed opportunities to maximize synergies between various actors that impact health [2]. While rigorously measuring the extent that global health initiatives strengthen health systems and increase capacity is methodologically fraught with challenges and limitations, there does seem to be overwhelming and increasing evidence that we as a global community can do better [3-6].

Enhancing the capacity of local organizations, strengthening the interactions between them, and improving institutions that underpin these interactions are critically important in order to build strong health systems. While the terms “organizations” and “institutions” are often used interchangeably, we refer to institutions as “rules of engagement between stakeholders.” Institutions are critically important for effective governance within health systems.

Multiple organizations interact to influence health systems, including ministries of health, public health organizations, health facilities, private sector players, universities, health policy institutes, non-governmental organizations, professional associations, and community-based organizations. These organizations perform a range of functions that directly or indirectly affect health, for example: identifying current and emerging health challenges; prioritizing needs; making and analysing policy; performing research; organizing social movements; providing services; promoting health behaviour change; and training health professionals. ‘Learning organizations’ [7] which can respond to constantly changing health challenges and the evolving context shaped by demographic, epidemiological, political, legal, economic, social, and technological developments are critical ingredients for HSS [8-10].

Not all ’successful’ health organizations contribute to the strengthening of health systems. For example, organizations involved in activities that are labelled “health systems strengthening” with short-term goals and undue focus on narrow programmes, diseases, or populations, often do not give adequate consideration to strengthening the entire health system, despite being labelled as such [4,11]. Hence, health system leadership need to establish formal (for example contracts, laws, regulations, and policies) and informal (including codes of conduct, norms of behaviour, and conventions) institutions [12] to enable different organizations to learn, adapt, and interact in ways that improve population health in an equitable and efficient manner. This article discusses the need for increased emphasis on organizational capacity building in global health through a complex adaptive systems lens, using lessons learned from case studies. Our manuscript is one of the products of a Rockefeller Foundation-sponsored meeting on health systems and complex systems thinking in Bellagio, Italy in August, 2012, which had strong presence from leaders in low-income countries [13]. While our particular focus is on the role that donors can play, we recognize that there are a number of other stakeholders (governments, citizens, businesses, professionals, etc.) that can also apply these concepts to strengthen health systems. We begin our paper by summarizing key ideas from organizational, institutional, and complex adaptive systems theory that have informed our discussion.

### Organizational studies

Organizational studies is a broad, well-developed discipline, covering areas that affect health including leadership, team dynamics, culture, and institutional environment [14]. Despite potential for synergies, collaboration between health researchers and their organizational studies counterparts has been inadequate. There are seven main perspectives within the discipline, as identified by Currie, et al.: contingency, transaction costs, resource dependency, networks, ecology, institutional, and complexity.

The study of organizational learning provides unique insights into the ways that organizations evolve and interact with one another to strengthen health systems over time, as it addresses challenges of knowledge mobilisation by highlighting the practice-based view of knowledge, which is tacit and embedded in context. Improving performance, then, is less about the application of external or formal evidence, and more about leveraging practice-based knowledge held by health professionals on the frontline [15]. Good practice can diffuse from one part of the system to another as organizational learning ensues across organizations and professions within a national or regional system.

### Institutional theory

Institutions represent repeated patterns of behaviour, and may remain for decades not because they are the most efficient or equitable, but because the transaction costs of change (such as the financial cost of changing infrastructure, or the social cost of challenging existing norms and interests) are too high [12]. This permanence of structures, roles, and processes for prolonged periods of time (sometimes despite preferable alternatives) is known as path dependence and can stifle economic and health systems development, particularly when created to privilege the interests of particular groups in society. Path dependence, however, is not necessarily negative; developing effective institutions and organizations can create a virtuous cycle, creating incentives that align behaviour with development. The unmet challenge is in engaging in the political sphere to design mechanisms for implementing and embedding institutions that are transparent, socially and economically efficient and thus conducive to health systems development.

### Complex, adaptive systems lens

Social systems like health have been described as “complex, adaptive systems” (CASs) because challenges such as infectious diseases, non-communicable diseases, and the social determinants of health are continuously changing (often unpredictably), and they are affected by a diverse array of interconnected factors [16,17]. Health systems that respond to those challenges are also complex, as they are constrained or assisted by varied socio-cultural contexts. In addition, they attempt to deliver a variety of preventative and curative interventions, and consist of individuals and a multitude of institutions and organizations, each with its own immediate goals, objectives, and perspectives.

CASs are path dependent, self-organizing, and composed of diverse, interconnected subsystems. They can be analysed using methods such as concept mapping; social network analysis; system dynamics modelling; program budgeting and marginal analysis; and the tools for knowledge management and translation [18]. Organizations in CASs should have a large degree of autonomy equipped with “learning ability”, a working environment which integrates knowledge management and encourages innovations. Top-down, command and control, blue-print strategies should be avoided when health challenges and interventions are complex to minimize unintended consequences.

## Discussion

### How do institutions influence local organizations and health systems in low-income countries?

While institutions can enable organizations to realize their potential to strengthen health systems, all too often they force organizations to adopt inefficient behaviours, which stifle learning and development. Inadequate capacity, ineffective enforcement of policies and regulations, lack of accountability, and weak monitoring systems may reinforce such inefficient behaviours. Additionally, inefficient behaviours may be created by sudden influx of new funding, which alters existing rules of engagement among local organizations. National norms and behaviours, in particular, influence organizations and their ability to strengthen health systems by creating an environment that fosters local engagement and learning. Examples of these norms and policies, and other institutions that enable and constrain the ability of organizations in low-income countries to strengthen health systems can be categorized as global, national, or organizational institutions.

On a global level, institutions in low-income countries are often focused on short-term, disease specific interventions that lack the sustainability required for strengthening health systems. Macro-level global policies, such as structural adjustment, intellectual property issues, and pharmaceutical pricing, impact organizational capacity. Organizations in low-income countries such as ministries of health, universities, and health policy institutes are chronically underfunded, and funding is too often unpredictable and exclusively for short-term projects. Piecemeal funding can lead to conflicting personal incentives, such as expert consultants that focus on short-term objectives at the expense of building capacity. Barriers caused by the funding process are heightened by monitoring and evaluation of policies and practices. Target indicators are often too rigid, short-term, and simplistic to allow for longer-term capacity building. A lack of coordination and cooperation between global health donors has too often led to inefficient and redundant reporting systems, and fragmented activities. Human resource policies become a constraining institution when external technical and other experts do not develop sustainable capacity and empower local people. Capacity building practice norms on a global level emphasize building individual technical capacity (often related to the clinical delivery of disease specific treatment and services), rather than institutional and organizational capacity.

At the national level, health policies, social norms, and private sector influence strongly affect the capacity of health systems. National health policies such as market policies in health, insurance policies, regulation, financing and payment schemes (user fees), allocation of funds (selective primary healthcare), etc. enable or impede health organizations’ capacity to deliver health interventions effectively. Some social norms such as solidarity, participatory decision making, and treating health and wellness as a priority at the individual, family, and community level can lead to stronger health systems, as they enable individuals and organizations to engage in effective transactions and plan for the future with relative certainty. Eliminating social norms with negative effects such as corruption, theft, informal payments, and nepotism, can also strengthen health systems. Furthermore, the private sector influences national policy and also plays a significant role in the provision of health care and health supplies.

Organizational institutions are also crucial in strengthening health systems. Interorganizational norms and practices, such as a shared vision of the future that includes commonly held goals and objectives, can lead to communication channels and collaborative relationships between organizations. Institutions that strengthen mutual trust, teamwork, role clarity, and appropriate incentives are needed. Commonly held expectations encourage shared learning between organizations that can lead to dissemination of innovative ideas and processes, and scaling-up of effective interventions. Intraorganizational social norms, including gender discrimination and the ability to challenge hierarchy, can impact health organizations’ ability to deliver health interventions effectively, learn, and adapt. Mechanisms to ensure regular, timely feedback are needed to ensure ongoing learning from experience, including mistakes and unintended consequences. Health organization managers too often lack adequate accountability. Finally, remuneration and budgetary allocation policies need to be structured to facilitate systems strengthening, or at the very least not to create perverse incentives to undermine such objectives.

### What types of collaboration between global health actors and organizations work best to strengthen health systems?

There is no one-size-fits-all way of maximizing synergies between various actors to strengthen health systems because local contexts vary significantly. Well documented shortcomings of the so-called “blue-print” model [19] have not prevented donors from frequently applying it in global health practice — where the donors and ‘external experts’ identify a need (such as a specific disease) in a given population, decide on interventions, and outline implementation plans, all with limited input from local stakeholders. Such top-down models do not adequately consider the unpredictable ways challenges in complex systems such as health emerge, and the ways societies, organizations, and professionals respond and adapt to these challenges [16,17,19]. By contrast, “bottom-up” social movements more often help achieve sustainable political and social change [20].

A recent study of health systems that have achieved ‘good health at low cost’ identified several lessons in relation to factors that improved the success of HSS efforts [21]. These lessons include the articulation of a vision and long-term strategy; consideration of the constraints imposed by path dependency; building consensus at a societal level; allowing flexibility and autonomy in decision making; resilience – learning from experiences, feeding back into the policy cycle; support from the broader governance and socioeconomic context in the country; being in harmony with culture and population preferences; achieving synergies among sectors and actors; and demonstrating openness to dialogue and collaboration between public and private sectors, with effective government oversight [21].

It remains unclear, however, how these successful conditions arise, and the role played by local organizations and institutions in the development of those conditions. We contend that viewing social and organizational change through the lens of complex, adaptive systems provides unique insights into such change [19]. Policies and procedures often exist not because they are the most efficient, but because of historical antecedents and high current transaction costs, a phenomenon described as path dependence. Change often happens not when detailed plans are dictated from the top down, but when on-the-ground agents self-organize to maximize the equity and efficiency of health actions locally. When systems and organizations are complex, the greatest need is often an environment where locals can self-organize to develop innovative and context appropriate approaches to health challenges through local organizations and networks.

Through our deliberations, we have identified three case studies (the Health Systems Trust in South Africa, the African Center for Global Health and Social Transformation in Uganda, and the Health Policy Analysis Unit and Health Policy Analysis Center in Kyrgyzstan) that highlight important interactions between local organizations and institutions, and offer concrete examples of the complex, iterative process of HSS described above.

The Health Systems Trust (HST) in South Africa was established during the final years of the apartheid regime to support the positive transformation of the health system [22]. It emerged out of negotiations with the African National Congress and donors who recognized the need for a new organization that could assist the post-apartheid government with health systems research and health systems policy development. As a non-government organization with an independent Board of Trustees, it was designed to work with and through the different health systems structures. These included the national and provincial ministries of health, the parliamentary health portfolio committee, and academic health departments. HST provides an example of an organization that receives feedback from a variety of sectors and organization, helping to maximize coordination.

HST’s functions include: funding strategic policy research studies; acting as a broker and bridge between research production and policy formulation; systematically working with public health management structures to overcome the bottlenecks and barriers in the way of policy implementation; acting as a conduit for the flow of information between the top and the ground-level of the health system; producing an annual report on health improvement and health systems performance; working to improve the coverage of health issues in the media (print, radio, and TV); and playing a critical ‘facilitatory’ role. It has been highly flexible and able to respond to emerging challenges, as well as being able to think strategically with a five-year time frame in mind. HST has retained a degree of independence that has been critical for it to play the multiple roles described above by diversifying its funding sources, with funds coming from the government and other donors.

The African Center for Global Health and Social Transformation (ACHEST) is an initiative of a network of African and international leaders in health and development to promote evidence-based, technically sound policies and strategies that are owned and driven by African populations themselves [23]. The organization functions as an independent think tank and network that forges alliances and partnerships with individuals and organizations within Africa and around the world. As such, ACHEST is an interesting study of an organization whose focus is on systems-level interactions between organizations that impact health. It conducts policy and strategy-oriented research focused on Africa's engagement with global partners in health, economic, and social development. ACHEST also promotes and advocates for the development of capacity of African professionals and institutions to pursue excellence and engage as leaders and active change agents in their communities, countries, and the global arena. The organization is currently working on mapping key health systems organizations in Africa and their role in strengthening health systems.

A collaboration between the Kyrgyzstan Ministry of Health, the World Health Organization, and the UK Department for International Development (DFID) serves as an interesting case study of collaboration between organizations, as well as the need to adapt institutional arrangements based on varied contexts [24]. A team of professionals organized as a Health Policy Analysis Unit was initially formed to create demand for political health analysis and evidence-based policy making. Their activities included providing recommendations in published reports. While initially formed as an autonomous public body under the Ministry of Health, stakeholders soon realized that there were managerial obstacles associated with its association with the ministry, and the need for increased independence. As such, after four years, a new (but closely related) organization, the Health Policy Analysis Center, was created as a public foundation that was not directly affiliated with the government, but maintained its close association by having several Ministry of Health members on its Steering Committee. An independent report demonstrated several key factors that contributed to this successful collaboration between an external funder, local health organizations, and institutional arrangements: an unusually long timeline (11 years); a close working relationship between partners; and a flexible approach to management by donors.

The three case studies described above highlight many organizational, institutional, and complexity concepts described previously, such as consideration of how organizations interact; ensuring adequate coordination; and adaptability to evolving environments. More specifically, these cases highlight a key message from complex adaptive systems: innovative and context-appropriate health solutions are more likely to emerge from self-organization of locals when organizations are given the freedom to adapt to local needs. Below we discuss two concrete lessons that deserve special attention as donors consider how to approach increasing organizational capacity with a complexity lens: the need for donors and recipient organizations to work as equal partners, and the need for leadership in low-income countries.

### Need for donors and recipient organizations to work as equal partners in improving institutions that strengthen health systems

Although the importance of supporting local organizations in HSS activities is recognized, this recognition has yet to adequately translate into policies to influence investments or actions [25,26]. The Paris Declaration and other similar documents have established principles of country ownership, alignment, harmonization, results focus, and mutual accountability in order to improve aid effectiveness and donor-recipient country engagement [27]. However, in practice the principles are not always adhered to – in particular, weak capacity of countries is used as an excuse to limit country ownership [28]. Instead, too often donors stress a blue-print model that strengthens technical capacity in one part of the system, rather than enhancing system-wide governance or “soft” organizational capacities – such as communication, trust building, diplomacy, networking, making sense of complex social situations, political advocacy, and leadership – that are critical for improving institutions and systems [29]. The so called soft capacities are highly dependent on social relationships and shaped by evolving context, and require time (sometimes decades) to develop, but are important for addressing the donor-recipient country power asymmetries and developing equal partnerships among stakeholders involved in strengthening country health systems—donors, external experts, managers, and local communities.

### Setting the HSS agenda: leadership in low-income countries

An increasingly dynamic and interdependent post-MDG world requires new ways of working to improve global health, underpinned by complex systems thinking and building local capacity [16,17,30-32]. In low-income countries, the transformation of institutions to support local organizations that strive to strengthen health systems will require visionary and courageous health leaders who can stress the need for strong organizations and institutions, network with others to enhance shared learning, and collaborate with external donors and experts as needed to create and support learning organizations. There is always tension between the top-down, more individualistic variant of leadership for transformational change, and the need for its spread leadership across organizations and professions to sustain such change. The challenge is to pluralize leadership, from the initiator of transformation to others, but to do so in a way that ensures the spread of leadership is channelled, rather than contested, for a coherent, lasting effect [33].

A consensus statement from our recent Rockefeller Foundation-sponsored Bellagio meeting on health systems and complex systems thinking highlighted the need for local leadership to strengthen health systems by increasing local organizational capacity (see the ‘Bellagio consensus statement’ subsection) [13]. There are examples of such approaches being successfully implemented to varying degrees around the globe. For example, strong ministerial leadership in countries such as Rwanda and Ethiopia, with support from external donors, has resulted in national ownership of development plans and increased donor coordination [34]. Health reforms in Kyrgyzstan provide another good example of such collaboration [24]. In addition, a number of initiatives have emerged recently that support local health leaders, including Strong Ministries for Strong Health Systems [35], Emerging Voices for Global Health [36], and the Consortium for Health Policy and Systems Analysis in Africa (CHEPSAA) [37]. At our Bellagio meeting and through related discussions, we also identified a number of questions for research and exploration in order to better understand factors that that lead to successful organizational capacity for HSS (See the ‘Important questions for future research and exploration’ subsection).

#### Bellagio consensus statement: use of a complex adaptive systems lens for health system strengthening [13]

A group of 22 health professionals and stewards, representing 15 countries, gathered in Bellagio, Italy from August 27–31, 2012, concerned about the attainment of the highest possible level of health as a most important world-wide social goal whose realization requires universal health coverage, as well as the action of many other social and economic sectors in addition to the traditional health sector. Central to achieving this goal are robust and dynamic health systems. After our discussions and deliberations, we agreed upon the following seven points, which we believe warrant serious consideration at all levels of global health practice, research, policy, and education:

1. Health systems are complex adaptive social systems (CAS) that are comprised of a wide variety of people, organizations and networks, each with its set of values and interests that must be aligned to achieve health and other health systems goals.
2. Health systems strengthening (HSS) has emerged in recent years as central to promoting and protecting health and relieving suffering. HSS is a complex, iterative, and learning process wherein the interactions between actors, structures, services, and subsystems are optimized over time while striving for health systems goals. As such, this process:
  Is highly contextual and influenced fundamentally by institutional relationships at local, national, and global levels, and will differ greatly from country to country and within countries at different times.
  Involves people and organizations outside of what is generally thought of as a health system, including health-related sectors; the private sector; agriculture; education; and others.
3. The process of health systems strengthening depends fundamentally on the ability of in country organizations to learn over time; adapt to emerging challenges; and optimize interactions with citizens, communities and other organizations to reach health systems goals. These organizations should become “learning organizations,” organizations that are continuously expanding their capacity to create their own future [7]. While technical capacity is very important, other capacities are also key, e.g.:
  Prioritizing Needs
  Taking Risks
  Mobilizing
  Advocating
  Identifying and Supporting Participatory Leadership
  Maximizing Synergies Between Sectors and Disciplines
4. The relationship between external global players such as donors and in-country organizations has been dominated by a focus on short-term, results-driven actions. Such a focus too often undermines local organizational capacity and leads to fragmented efforts.
5. We as a global health community can do much better to strengthen organizational capacity that leads to strengthened health systems around the world.
6. The CAS approach deserves serious consideration and testing in terms of its application to organizational capacity building for HSS because of its analytical and transformational potential. Key CAS themes in social systems have been neglected in much of health systems activities, such as:
  Collaboration Across Sectors and Disciplines Around a Shared Vision
  Feedback Loops between Interconnected Components
  Social and Organizational Networks
  Transformational, Systems Level Leadership at all Levels
  Ongoing, Iterative Learning
  Creation of a Local Environment that Encourages Emergent Self-Organization and Innovation
7. We hope that all global health stakeholders from practice, academia, policy, and education will consider the points above, and make appropriate changes within their sphere of influence to increase local organizational capacity and strengthen health systems around the world.

#### Important questions for future research and exploration

1. What role do disease-specific initiatives play in an approach to HSS that focuses on enhancing organizations and improving institutions?
2. More rigorous case studies are needed to highlight successful and unsuccessful approaches to enhancing organizational capacity and improving institutions, and refine recommendations. Which methods and measures will accelerate shared learning across jurisdictions?
3. How can we best integrate systems sciences, organizational studies, and institutional analysis approaches and methods into health education, policy, practice, and evaluation?
4. How can we encourage and support future health leaders that will establish and maintain organizational learning in low-income countries?
5. Why have some countries (such as Thailand and Brazil) developed organizations that have been relatively more effective in shaping policies towards HSS as compared to others (such as India and South Africa)?
6. How have specific donors been engaged in organizational and institutional strengthening?
7. How might donors and recipient organizations more fully engage as equal partners while exercising mutual respect in prioritizing needs, developing plans, implementing interventions, and modifying approaches based on changing environments?
8. How can institutional frameworks [38], toolkits [39], and approaches be better applied to improve interactions between global actors and local organizations to strengthen health systems?
9. How do we measure and attribute the contributions made by organizations, institutions, and donors to the effectiveness of health systems?
10. How can we create institutions that ensure effective dialogue and collaboration between public and private sectors, with effective government oversight?

## Summary

New ways of thinking and working, underpinned by complex adaptive systems and building local organizational capacity, offer the potential for developing sustainable and people-centred health systems as we seek to define the post-MDG agenda. However, there is a need to graduate from a primary focus on reductionist approaches that emphasize a single disease, and instead direct efforts to systems approaches that improve institutions and help enhance organizational capacity in countries, as well as develop local leadership and innovative solutions. Only then can we expect to develop strong health systems that can efficiently and equitably address the disease burden faced by low-income countries.

## Abbreviations

HSS: Health systems strengthening
MDG: Millennium development goals
CAS: Complex adaptive system
HST: Health systems trust
ACHEST: African Center for Global Health and Social Transformation
DFID: UK Department for International Development
